# Are we assessing medical students' recall or readiness for practice?

**DOI:** 10.3389/fmed.2026.1801030

**Published:** 2026-04-09

**Authors:** Waseem Jerjes, See Chai Carol Chan, Azeem Majeed

**Affiliations:** Department of Primary Care and Public Health, Imperial College London, London, United Kingdom

**Keywords:** assessment dashboards, clinical competence, longitudinal assessment, medical education, medical student assessment, programmatic assessment, readiness for practice

## Introduction

Medical education is full of apparent certainties. Pre-clinical students can memorize enzyme pathways, rare syndromes, and lists of adverse drug reactions well enough to perform strongly in single-best-answer (SBA) examinations. Yet the transition from student to newly qualified doctor often exposes a more uncomfortable reality: clinical work is not a rehearsal of tidy facts but an ongoing negotiation with uncertainty, time pressure, competing risks, incomplete information, and the needs of other people in a system.

The problem is not that knowledge is unimportant. The problem is that much of what we currently reward and amplify is a narrow and unreliable proxy for readiness to practice. When assessment culture predominantly prizes rapid recall of decontextualised details, learners logically optimize for what is rewarded. Students build their weeks around question banks, flashcards, and revision strategies that primarily target short-term exam performance. Surveys of learner assessment preferences repeatedly show that the methods learners perceive as “fair” or “efficient” are often those that best align with predictable recall demands rather than authentic practice performance (1–5). That alignment is reinforced by an expanding industry of algorithmic learning platforms that personalize drills and predict forgetting, further intensifying the message that the main task is to memorize more, faster.

The educational consequences are visible. When learners rely heavily on rehearsal of disclosed items or narrow patterns of remembered answers, their conceptual grasp becomes fragile—especially when they face new presentations that share surface features with familiar cases but differ in clinically important ways. Interventions that shift learners away from rote memorisation toward conceptual understanding can produce measurable improvements, precisely because memorisation-based performance can mask deeper misunderstandings until clinical complexity forces them to the surface (2, 6–12). The move from “exam-ready” to “practice-ready” should not feel like cognitive whiplash; yet it often does.

This mismatch is not simply pedagogical. It reflects the collective choices medical schools and regulators make about what they count, what they tolerate, and what they expect learners to become. If assessment drives learning, then assessment design is not a technical afterthought—it is the curriculum.

## The assessment culture problem: what we reward becomes what we teach

SBA examinations are widely used because they are scalable, reliable, and accepted. They are also relatively inexpensive compared with many performance-based assessments. But reliability and convenience are not enough. The central question is whether our dominant assessment methods validly sample the capabilities that constitute safe, effective clinical work.

A useful lens is van der Vleuten's utility formula, which frames assessment quality as the product of reliability, validity, educational impact, acceptability, cost, and feasibility (3). SBA formats can score highly on reliability, acceptability, and feasibility, and well-constructed SBA items can assess applied reasoning when they are carefully blueprinted and written to test interpretation rather than simple recognition. However, validity and educational impact still depend on item quality and the degree to which questions require meaningful application rather than recognition of rehearsed fragments. Reviews applying utility-based approaches to SBA item sets highlight the practical challenge: many assessments drift toward what is easiest to construct and mark, rather than what best captures real clinical judgement (4). When that drift occurs, assessments may still look rigorous but function educationally as memorisation incentives.

Even when we add oral examinations, vivas, and Objective Structured Clinical Examinations (OSCEs), we do not automatically solve the validity problem. Performance assessments can introduce their own distortions when scoring is influenced by factors unrelated to the construct being measured. Construct-irrelevant variance is well described in high-stakes testing, including effects of examiner strictness, interactional style, and other factors that can bias outcomes while appearing “objective” (5). Evidence from OSCE settings suggests that examiner seniority and experience can be associated with bias in certain domains such as communication scoring (6). Language and accent effects also matter: if examiner judgements shift with a candidate's accent, fluency, or linguistic style, then the assessment is partly measuring communication presentation rather than clinical competence (7, 13–17). In practice, these effects can reward confident delivery and penalize cautious reasoning, even when the latter is safer.

In short, the problem is not the existence of SBA assessment itself, but a culture in which recall-heavy written formats and performance under artificial conditions are allowed to stand in disproportionately for readiness. This is especially problematic when the transition to practice requires not just knowing, but knowing how to act when knowledge is incomplete.

## Knowledge mastery is not memory performance: designing “desirable difficulties” for durable, usable knowledge

If we accept that knowledge matters, we must become more precise about what kind of knowledge we want learners to have. Clinical knowledge is not simply stored; it must be retrieved under pressure, adapted to the person in front of you, and integrated with constraints and values. That means medical education should differentiate between short-term performance and durable learning that transfers.

The learning science concept of “desirable difficulties” offers a pragmatic route forward. Strategies such as spaced retrieval, interleaving, and effortful recall can improve long-term retention and transfer by strengthening memory in ways that mimic real use (8, 18, 19). Importantly, these strategies are not just about remembering more; they are about remembering in a form that supports discrimination between similar presentations, adaptation to context, and resistance to the illusion of competence created by cramming.

In practical terms, this suggests an assessment culture that privileges repeated, low-stakes retrieval over isolated, high-stakes recall events. It also suggests that the most meaningful learning signal is not whether a student can reproduce information once, but whether they can retrieve and apply it weeks later, in slightly altered circumstances, with competing priorities.

However, learning strategies alone cannot overcome an assessment regime that continues to pay primarily for short-term recall. If we want students to engage in spaced, cumulative learning, we must build a system where cumulative evidence matters more than a single performance spike.

## Programmatic assessment: shifting from snapshots to longitudinal judgements of competence

Programmatic assessment aims to replace single, high-stakes “snapshots” with many smaller data points collected over time, triangulated with narrative feedback, and used to support both learning and judgement (9). The promise is not that every micro-assessment is perfect, but that a programme of assessment—properly designed—produces a more valid picture of competence than any single tool.

International experience of implementation shows both the appeal and the difficulty. Programmatic assessment requires not only cultural change but operational infrastructure: protected faculty time for observation and feedback, administrative support for scheduling and record-keeping, agreed formats for narrative comments, and reliable processes for collating evidence across hospital and community placements. Institutions also need clear governance structures, including who reviews aggregated evidence, how disagreements between assessors are handled, how concerns are escalated, and what level of evidence is sufficient for progression decisions. These requirements increase workload during implementation, especially in distributed programmes where multiple supervisors contribute data under service pressure, but they are necessary if longitudinal judgement is to be credible, fair, and educationally useful (10, 20–22). A feasible starting model may therefore be phased rather than wholesale, using a limited number of recurring assessment activities, shared rubrics, scheduled review points, and a trained decision-making group before broader expansion. Yet those challenges are also the point. Readiness for practice is inherently longitudinal. No single exam sitting can credibly represent a learner's ability to practice safely across contexts, teams, and uncertainty levels.

A programmatic approach also creates space to reward the “invisible work” of clinical practice: how learners prioritize, how they recognize risk, how they use resources, how they communicate uncertainty, and how they modify their plan when the situation changes. These are precisely the capabilities that traditional recall-heavy assessments under-sample. Done well, programmatic assessment can also improve fairness. It reduces the influence of one-off performance fluctuations and shifts decision-making toward patterns, trends, and consistency across contexts. It can reduce the dominance of construct-irrelevant variance by relying less on a single oral performance and more on repeated sampling of comparable constructs. Crucially, it can support the message that looking things up and asking for help are not admissions of incompetence but elements of safe practice.

## Assessing safe resource use: from “closed-book medicine” to authentic verification in real time

Modern clinical practice is resource-rich. Clinicians consult guidelines, formularies, pathways, colleagues, and increasingly decision-support tools. Yet many assessments still act as if safe practice is primarily an internal memory performance.

A more defensible model is to explicitly assess resource use: how learners locate information, judge its trustworthiness, apply it to the patient's context, and document their reasoning. National assessments have already demonstrated that large-scale evaluation of applied prescribing capability is feasible. The UK Prescribing Safety Assessment provides an instructive example of assessing prescribing competence at scale, including decision-making that mirrors real prescribing tasks rather than pure recall (11). The broader implication is straightforward: if we can assess safe application in prescribing, we can design similar resource-permitted tasks in other domains, including diagnostic reasoning, escalation decisions, and safety-netting.

This is also where the realities of AI must be handled with maturity. Learners already use AI-enabled tools for study, summarisation, and question generation. The appropriate educational response is not to pretend this is not happening, but to define explicitly what is being assessed: verification, contextualization, justification, and safe decision-making. In other words, the assessed skill becomes not “producing an answer unaided” but “arriving at a safe decision through appropriate methods.”

## Tolerance of “not knowing”: uncertainty as a competency rather than a defect

A distinctive feature of clinical practice is that uncertainty is not the exception; it is often the default. A curriculum that trains learners to behave as if certainty is always available can inadvertently teach two unsafe behaviors: false certainty (acting as if the answer is known) and paralyzing uncertainty (not acting when action is needed).

There is a growing argument for making uncertainty an explicit curricular and assessment domain, including teaching learners how to work with ambiguity, weigh probabilities, and plan next steps when information is incomplete (12). This is not about celebrating indecision. It is about teaching safe uncertainty: a stance where the clinician can articulate what is unknown, identify what information would change the plan, and implement safety-netting and escalation strategies.

The idea of “safe uncertainty” has long been described as a practical clinical position—neither rigid certainty nor anxious indecision, but a disciplined and communicable way of working with what cannot be fully known (13). Translating that into assessment means designing tasks that deliberately include incomplete data, evolving information, or competing risks, and then scoring not only the final diagnosis or plan but the quality of uncertainty management. At an earlier stage, a learner might simply identify what remains uncertain; at a more advanced stage, they should be able to state a working diagnosis, assign a confidence level, identify what information would change the plan, and specify clear review or escalation triggers. That includes stating confidence levels, setting review triggers, documenting thresholds for escalation, and communicating the plan to patients and team members.

A key equity advantage also emerges here. If we normalize “looking it up” and “here is how I will close the gap,” we reduce the unfair advantage that may accrue to those who can perform fluent recall under observation, and we align assessment with what clinicians actually do.

## Adaptive expertise: assessing the ability to adjust when the pattern does not fit

If the future clinician's main task were only to reproduce learned responses, memorisation would be enough. But clinical work frequently demands adaptation: to atypical presentations, multimorbidity, polypharmacy, resource constraints, and patient preferences. This is where the concept of adaptive expertise becomes essential. Educational approaches that promote adaptive expertise aim to develop clinicians who can apply routine solutions efficiently when appropriate, but also innovate, reframe, and adjust when routine approaches fail (14).

Assessments should therefore sample adaptation, not just reproduction. The cognitive psychology distinction between fast, intuitive pattern recognition and slower, analytical reasoning provides a useful structure for designing such sampling. Clinical reasoning often moves between rapid impressions and deliberative checking; safe practice depends on knowing when to shift mode, especially when initial patterns are misleading (15). Learners should be assessed on their ability to recognize when a case does not fit the expected pattern, to slow down, to test alternative hypotheses, and to revise decisions as new information emerges.

This is not an argument against pattern recognition; it is an argument for making the switching behavior visible and assessable. Key-features problems, evolving-data cases, and short “reasoning notes” can capture whether a learner is anchored to a first impression or can adjust appropriately under constraint.

## Principled adaptation and contextualized care: what “good” decisions look like in the real world

Even when knowledge is robust and uncertainty is managed safely, clinical decisions are rarely “purely technical.” They take place within context: the patient's goals, the family's capacity, the service's constraints, and the ethical trade-offs involved.

Educational interventions are more likely to create meaningful impact when they recognize that outcomes emerge through layered mechanisms, not simple input-output relationships. The concept of “principled adaptation” is helpful here: rather than judging whether a learner replicates an idealized model answer, we can judge whether they adapt decisions responsibly while staying anchored to sound principles (16). This matters because many real clinical decisions involve choosing among imperfect options, balancing competing harms, and making time-sensitive compromises.

Contextualized care is not a soft add-on; it is a measurable clinical competency. The ability to integrate contextual factors—such as socioeconomic constraints, health literacy, competing priorities, and system barriers—can be assessed and should be viewed as part of competence, not merely professionalism (17). This has direct implications for assessment design. If we continue to score primarily for recall and “standard answers,” we risk training clinicians who can pass exams but struggle to deliver appropriate care when context alters risk, feasibility, or acceptability.

A more authentic assessment culture would therefore reward (a) explicit reasoning, (b) safe uncertainty practices, (c) appropriate resource use, and (d) contextualization of care decisions. These elements align with what patients actually experience as quality and safety.

## Discussion

This concern is also supported by the broader preparedness-for-practice literature. Reviews of newly qualified doctors consistently identify gaps not in all domains, but in precisely those areas that depend on applied, situated performance: prescribing, clinical reasoning, acute care, prioritization, handover, and navigating the ward environment (18, 19). Seen in that light, the issue is not that graduates know nothing; it is that our assessment systems can over-certify recall while under-sample the work of early practice.

The solution is not to “abolish memorisation,” discard SBA examinations altogether, or lower standards. Clinical knowledge is foundational, and well-designed knowledge-based formats still have an important place. The issue is proportionality and alignment: we must retain rigorous expectations for knowledge while ensuring that the assessments that drive learning also sample the capabilities that make knowledge usable and safe in practice.

A coherent shift would include four linked changes.

First, we should treat durable, usable knowledge as the target, not short-term recall performance. That means systematically embedding spaced retrieval, interleaving, and cumulative low-stakes testing as normal educational infrastructure, consistent with the evidence on desirable difficulties (8). Where high-stakes assessments remain necessary, they should be balanced by longitudinal signals that discourage cramming and reward sustained learning.

Second, we should adopt programmatic assessment principles, not as a fashionable slogan but as an explicit strategy for increasing validity. Many small samples of performance—quizzes, prescribing tasks, reasoning notes, supervised clinical encounters, and structured reflections—can be combined to produce a richer judgement of competence than any single exam sitting (9). Implementation is challenging, and international experience shows that feasibility depends on more than educational intent. Programmes need governance, faculty development, protected staff time, and practical systems for scheduling observations, storing evidence, and reviewing it consistently across settings (10, 20–22). Longitudinal evidence must also be governed explicitly: low-stakes data can support coaching, but high-stakes progression decisions should rest with a trained group that reviews aggregated evidence over time, applies clear decision rules, and attends deliberately to bias and fairness (20–22). There is no single blueprint that every school can lift unchanged, but the operational questions are unavoidable: which assessment tasks are worth collecting, how often they will be sampled, who will review them, and what institutional support will make the process sustainable. Yet the challenge is justified by the stakes: we are certifying readiness for practice.

Third, we should explicitly assess safe resource use and verification, rather than continuing the fiction that closed-book memory performance is the gold standard of clinical readiness. Prescribing assessments show that applied competence can be assessed at scale (11). Similar models can be developed for guideline use, risk stratification, escalation thresholds, and safety-netting. In a world where information is accessible, the key skill is not hoarding facts but using them responsibly.

Fourth, we should make uncertainty management and contextualization explicit assessment targets. Uncertainty deserves a formal place in curricula (12), and “safe uncertainty” should be treated as a clinical stance that can be taught, practiced, and assessed (13). Assessment should therefore reward not only the right answer when certainty is available, but the ability to recognize, monitor, and safely manage what remains uncertain. Making learners commit to a judgement, declare confidence, and then respond to corrective feedback can also turn error into a productive learning signal rather than something to conceal (23). Similarly, contextualized care should be recognized as an essential competency, with structured ways to evaluate whether learners can integrate context into decision-making (17). This is not about being “nice”; it is about making decisions that are actually safe, effective, and feasible for real patients.

One practical way to bring these elements together is to use a small set of recurring assessment activities that generate comparable data over time and collate them in a portfolio summary or learner dashboard (Table 1). Existing descriptions of learner performance dashboards in medical education, together with reports of dashboard use alongside longitudinal coaching and learning planning, suggest that their value lies not in sophisticated analytics, but in making multiple low-stakes data sources visible in one place for longitudinal review by learners and faculty (24, 25). In practice, each entry could be date-stamped, linked to a domain of competence, and accompanied by both a brief rating and a short narrative comment—for example, a retrieval quiz score, a reasoning note, a prescribing task, or an uncertainty statement gathered during routine teaching. Those data could then be reviewed at defined intervals by the learner and supervisor for coaching, and later by a trained group when progression decisions are required, with attention to trends, recurring concerns, missing evidence, and discrepancies between settings. The purpose is not surveillance; it is to make patterns interpretable across time so that judgement rests on accumulated evidence rather than isolated impressions. Seen this way, the dashboard can be as simple as a structured spreadsheet or portfolio page; what matters is consistent data collection, visible aggregation, and a clear process for how the information will inform feedback and decision-making. Table 1 illustrates the kinds of low-burden entries that could populate such a system, alongside the coaching prompts and learner follow-up actions that help convert data into feedback.

**Table 1:** Illustrative routine assessment activities and example outputs that can be collated longitudinally in a portfolio summary or learner dashboard to sample readiness for practice in routine teaching.

Data source	Suggested frequency	What it captures	What to look for when marking	Teacher coaching prompt	Student follow-up
Five-minute retrieval quiz (mixed topics)	Weekly	Retention, spaced practice effect	Accuracy over time; ability to explain why an answer is right/wrong	“Where did forgetting occur—what will you schedule next?”	Add weak items to personal spaced-practice deck; re-test next week
100-word reasoning note after a case	1–2 per clinic	Differential breadth, prioritization, thresholds to act	Alternatives considered; cues that would change plan; safety-net	“What would make you change your mind sooner?”	Write a one-line trigger list for the next similar case
Evolving-data OSCE/minicase (new info mid-station)	Monthly	Switching between rapid and analytical reasoning; responsiveness to change	Updates hypothesis/plan when data change; avoids anchoring	“What was the pivot point—and why?”	Note one anchoring trap and a counter-strategy
Resource-permitted prescribing task (BNF/NICE)	Fortnightly	Judicious resource use; application not recall	Finds guidance quickly; applies correctly to patient context	“Show me the bit you trusted—why that, not another?”	Save a snippet to a personal ‘go-to' library
Uncertainty statement with next steps (confidence + triggers)	Opportunistic	Safe uncertainty; risk management	States working diagnosis, confidence level, what remains unknown, and triggers to review or escalate	“What would make you change course, and by when?”	Document two escalation triggers and one step to close the gap
Key-features problem set (short vignettes)	Monthly	Decisions at critical steps	Gets the key decision right; justifies it briefly	“What single cue most influenced that choice?”	Add cue → action pairs to a pocket list
Mini-CEX: handover (SBAR)	Monthly	Communication, prioritization	Concise situation/risks; clear ask	“What risk did you make most salient, and why?”	Practice a 60-sec version; record and review
“Change-of-mind” log (reflection)	Ongoing	Cognitive flexibility; bias recognition	Specific moment of revision, not generic reflection	“What signal overcame your initial anchor?”	Write a pre-mortem for the next similar case

BNF, British National Formulary; NICE, National Institute for Health and Care Excellence; SBAR, Situation, Background, Assessment, Recommendation.

This approach also has a fairness argument. When we reward only speeded recall and polished performance, we risk disadvantaging learners whose strengths are reflective reasoning, careful verification, or who operate in a second language. A more authentic, longitudinal approach can better represent competence as it exists in practice: collaborative, resource-informed, and context-sensitive. Fairness, however, does not emerge automatically from adding more data; it depends on deliberate design, credible aggregation, and trained judgement (20–22).

Ultimately, the goal is alignment. If assessment drives learning, then our assessments must drive the kind of learning that prepares graduates for the day they first face a complex patient, a busy ward, and incomplete information. Readiness for practice is not a memory trick. It is the ability to retrieve knowledge under pressure, verify and apply it safely, communicate uncertainty responsibly, adapt when the pattern breaks, and contextualize care so that decisions work in the real world. These are assessable capabilities. The only remaining question is whether we are willing to make them count.

## References

[1] Kececi A Dogan CD Gonullu I. Assessment preferences of nursing and medical students: a correlational research. Nurse Educ Pract. (2022) 60:103305. doi: 10.1016/j.nepr.2022.103305 35144163

[2] Heber S Grasl MC Volf I. A successful intervention to improve conceptual knowledge of medical students who rely on memorization of disclosed items. Front Physiol. (2023) 14:1258149. doi: 10.3389/fphys.2023.1258149 37711461 PMC10499193

[3] Van Der Vleuten CP Schuwirth LW. Assessing professional competence: from methods to programmes. Med Educ. (2005) 39:309–17. doi: 10.1111/j.1365-2929.2005.02094.x 15733167

[4] Mirbahai L Adie J. Applying the utility index to review single best answer questions in medical education assessment. Arch Epidemiol Public Health. (2020) 1:1–5. doi: 10.15761/AEPH.1000113

[5] Haladyna TM Downing SM. Construct-irrelevant variance in high-stakes testing. Educ Meas Issues Pract. (2004) 23:17–27. doi: 10.1111/j.1745-3992.2004.tb00149.x

[6] Chong L Taylor S Haywood M Adelstein BA Shulruf B. Examiner seniority and experience are associated with bias when scoring communication, but not examination, skills in objective structured clinical examinations in Australia. J Educ Eval Health Prof . (2018) 15:17. doi: 10.3352/jeehp.2018.15.17 30016854 PMC6194481

[7] Kozato A Patel N Shikino K. A randomised controlled pilot trial of the influence of non-native English accents on examiners' scores in OSCEs. BMC Med Educ. (2020) 20:268. doi: 10.1186/s12909-020-02198-y 32799871 PMC7429462

[8] Bjork RA Bjork EL. Desirable difficulties in theory and practice. J Appl Res Mem Cogn. (2020) 9:475–9. doi: 10.1016/j.jarmac.2020.09.003

[9] Schuwirth LW Van der Vleuten CP. Programmatic assessment: from assessment of learning to assessment for learning. Med Teach. (2011) 33:478–85. doi: 10.3109/0142159X.2011.565828 21609177

[10] Torre D Schuwirth L Van der Vleuten C Heeneman S. An international study on the implementation of programmatic assessment: Understanding challenges and exploring solutions. Med Teach. (2022) 44:928–37. doi: 10.1080/0142159X.2022.2083487 35701165

[11] Maxwell SR Coleman JJ Bollington L Taylor C Webb DJ. Prescribing safety assessment 2016: delivery of a national prescribing assessment to 7343 UK final-year medical students. Br J Clin Pharmacol. (2017) 83:2249–58. doi: 10.1111/bcp.13319 28449302 PMC5595977

[12] Papanagnou D Ankam N Ebbott D Ziring D. Towards a medical school curriculum for uncertainty in clinical practice. Med Educ Online. (2021) 26:1972762. doi: 10.1080/10872981.2021.1972762 34459363 PMC8409968

[13] Mason B. Towards positions of safe uncertainty. Human Systems. (1993) 4:189–200.

[14] Pusic MV Hall E Billings H Branzetti J Hopson LR Regan L . Educating for adaptive expertise: case examples along the medical education continuum. Adv Health Sci Educ. (2022) 27:1383–400. doi: 10.1007/s10459-022-10165-z 36414880

[15] Kahneman D. Thinking, fast and slow. New York: Macmillan (2011).

[16] Cianciolo AT Regehr G. Learning Theory and Educational Intervention: Producing Meaningful Evidence of Impact Through Layered Analysis. Acad Med. (2019) 94:789–94. doi: 10.1097/ACM.0000000000002591 30640265

[17] Weiner SJ. Contextualizing care: an essential and measurable clinical competency. Patient Educ Couns. (2022) 105:594–8. doi: 10.1016/j.pec.2021.06.016 34158194

[18] Alexander C Millar J Szmidt N Hanlon K Cleland J. Can new doctors be prepared for practice? A review Clin Teach. (2014) 11:188–92. doi: 10.1111/tct.12127 24802919

[19] Michaelides A Mahr M Pydisetty G Loyala JV. Assessing the preparedness of foundation year 1 (FY1) doctors during the transition from medical school to the foundation training programme. BMC Med Educ. (2020) 20:106. doi: 10.1186/s12909-020-01999-5 32264871 PMC7137288

[20] Casiro O Regehr G. Enacting Pedagogy in Curricula: On the Vital Role of Governance in Medical Education. Acad Med. (2018) 93:179–84. doi: 10.1097/ACM.0000000000001774 28640029

[21] Ross S Hauer KE Wycliffe-Jones K Hall AK Molgaard L Richardson D . ICBME Collaborators. Key considerations in planning and designing programmatic assessment in competency-based medical education. Med Teach. (2021) 43:758–64. doi: 10.1080/0142159X.2021.1925099 34061700

[22] de Jong LH Bok HGJ Schellekens LH Kremer WDJ Jonker FH van der Vleuten CPM. Shaping the right conditions in programmatic assessment: how quality of narrative information affects the quality of high-stakes decision-making. BMC Med Educ. (2022) 22:409. doi: 10.1186/s12909-022-03257-2 35643442 PMC9148525

[23] Metcalfe J. Learning from Errors. Annu Rev Psychol. (2017) 68:465–89. doi: 10.1146/annurev-psych-010416-044022 27648988

[24] Boscardin C Fergus KB Hellevig B Hauer KE. Twelve tips to promote successful development of a learner performance dashboard within a medical education program. Med Teach. (2018) 40:855–61. doi: 10.1080/0142159X.2017.1396306 29117744

[25] Hauer KE Iverson N Quach A Yuan P Kaner S Boscardin C. Fostering medical students' lifelong learning skills with a dashboard, coaching and learning planning. Perspect Med Educ. (2018) 7:311–7. doi: 10.1007/S40037-018-0449-2 30187391 PMC6191394

